# Late spring urea application increased apparent carbon dioxide equivalence emissions but fall and summer applications did not

**DOI:** 10.1002/jeq2.70050

**Published:** 2025-07-07

**Authors:** G. W. Reicks, D. E. Clay, S. A. Clay, D. R. Joshi, J. Moriles‐Miller, S. Westhoff, S. A. Bruggeman

**Affiliations:** ^1^ Department of Agronomy, Horticulture, and Plant Science South Dakota State University Brookings South Dakota USA; ^2^ Department of Agronomy Kansas State University Manhattan Kansas USA; ^3^ Biology Department Augustana University Sioux Falls South Dakota USA

## Abstract

Synchronizing nitrogen (N) fertilizer application with plant N uptake, as opposed to applying earlier, is believed to improve N use efficiency while simultaneously reducing nitrate leaching, ammonia volatilization, and greenhouse gas emissions. However, little research has been conducted to confirm that this is true. Therefore, this study's objective was to determine the effect of urea application date (early fall, mid‐fall, late fall, late spring, early summer, and midsummer) and rate (0 and 224 kg N ha^−1^) on the apparent carbon dioxide equivalence (CO_2ea_). This completely randomized field experiment was conducted six times (dates) where each treatment was replicated four times. Soil temperature and moisture along with greenhouse gases (N_2_O, CO_2_, and CH_4_,) were measured every 4 h for 21 days at Aurora, SD, in 2017 and 2018. N_2_O and CH_4_ were expressed in carbon dioxide equivalence, and all three gases were summed to CO_2ea_. Delaying the fertilizer from early to late fall decreased CO_2ea_ from 69,375 to 18,420 g CO_2ea_ (ha × day)^−1^. During late spring through midsummer, CO_2ea_ were similar for the three application dates and averaged 101,500 g CO_2ea_ (ha × day)^−1^. This study showed that the lowest CO_2ea_ values for the 3 weeks following the application were associated with applying urea in the fall after the temperatures had decreased to less than 10°C or waiting until early summer. This research suggests that full‐season assessments of fall, spring, and summer urea application dates on CO_2ea_ are warranted.

## Core Ideas

- The apparent carbon dioxide equivalent (CO_2ea_) was calculated by summing the CO_2_, N_2_O, and CH_4_ emissions.
- Urea application increased CO_2ea_ during late spring.
- During spring and summer, soils had lower CH_4_ emissions but higher CO_2_ emissions than in fall.
- The CO_2ea_ following urea application needs to be confirmed with a full‐season assessment.

**Abbreviations**
CO_2e_
: carbon dioxide equivalence
GHG: greenhouse gas
SOC: soil organic carbon
WFPS: water‐filled pore space

## 1 INTRODUCTION

Nitrogen (N) fertilizer is a major input in nonleguminous crop production and a direct source of N_2_O emissions. However, in addition to its direct impact on N_2_O, it may impact carbon dioxide and methane (CH_4_) emissions, which have 273 and 28 times the global warming potential of CO_2_, respectively (Smith et al., 2021). Understanding the sources and mechanisms of these emissions can aid in reducing the direct and indirect impact of N fertilizer on greenhouse gas (GHG) emissions (Dong et al., 2018; Eagle et al., 2017; Nazir et al., 2024).

It is widely believed that applying fertilizer when N demand is highest will simultaneously improve N use efficiency while reducing nitrate leaching, ammonia volatilization, and N_2_O emissions (Graham & Bly, 2016; Thies et al., 2020; Venterea et al., 2016). However, research supporting this hypothesis is limited. The research that has been conducted suggests that interactions among the soil properties, climatic conditions, and the fertilizer application date and source can increase or decrease the carbon dioxide equivalence (CO_2e_) (Clark et al., 2020; Holman et al., 2024; Joshi et al., 2022; Thies et al., 2020; Venterea et al., 2010). For example, (1) Clay et al. (1990) showed that the treatment of urea with a nitrification inhibitor can reduce nitrate leaching while increasing ammonia volatilization, (2) Brugler et al. (2025) showed that splitting the fertilizer between preemergence and the corn (*Zea mays*) V6 growth stage may reduce N_2_O emissions prior to V6 and increase emissions after V6, and (3) Thies et al. (2020) demonstrated that applying urea in the fall to a silty clay loam soil when soil temperatures are below 10°C may have less ammonia (NH_3_) volatilization and N_2_O emissions than spring applied N. Results from these studies indicate that to optimize N efficiency and minimize losses, strategies that optimize timing need to be identified. One area of potential research is to compare fall, spring, and summer applied fertilizers on GHG emissions.

Fall applied fertilizer is of particular interest because fertilizer is often cheaper in the fall (Graham & Bly, 2016; Mueller et al., 2017). Unfortunately, little research has been conducted on the impact of fall applied fertilizer on the CO_2e_. Filling this gap involves conducting an initial screening of the possible fertilizer application dates followed by future research to conduct a full‐season assessment. Therefore, this study's objective was to determine the effect of urea application date (early fall, mid‐fall, late fall, late spring, early summer, and midsummer) and rate (0 and 224 kg N ha^−1^) on CO_2ea_.

## 2 MATERIALS AND METHODS

This is the third study based on the dataset described below. The first study (Thies et al., 2019) compared the static chamber approach (Venterea & Coulter, 2015) with the near continuous methods used in this study. The second paper (Thies et al., 2020) reported on the impact of fertilizer timing and rate on NH_3_, N_2_O, and CO_2_ emissions. This paper expands on this analysis to include CH_4_ and CO_2ea_.

The study site was located near Aurora, SD (44° 18′ 20.57″ N, 96° 40′ 14.04″ W), and it was on the border between the Bsh (semiarid) and DFa (continental wet all seasons) Köppen climate groups. The soil was a well‐drained Brandt silty clay loam (Fine‐silty, mixed, superactive, frigid Calcic Hapludolls) (Soil Survey Staff, 2018) on a 0%–2% slope. The parent materials at the site were loess (0–60 cm) over glacial outwash. The surface 15 cm contained 280 g clay kg^−1^ (28%), 65 g silt kg^−1^ (65%), 7 g sand kg^−1^ (7%), and 36 Mg ha^−1^ (1.8%) of soil organic carbon (SOC). This soil has a textural discontinuity between 60 and 80 cm (silty clay loam to gravel) that slows deep drainage (D) below the discontinuity to near zero during the growing season. The gravimetric water contents at field capacity and the wilting point were approximately 0.315 and 0.177 g g^−1^, respectively. Additional findings from this study site are reported in Clay et al. (2005, 1996, 2015).

This was a completely randomized study design in which urea was applied at two rates (0 and 224 kg urea‐N ha^−1^) in early fall (September 21, 2017), mid‐fall (October 11, 2017), late fall (November 1, 2017), late spring (May 1, 2018), early summer (May 22, 2018), and midsummer (June 12, 2018). Each treatment was replicated four times.

### 2.1 Site preparation and sampling intervals

Soybeans (*Glycine max*) were planted at the site in May 2017. To prepare the soil for the experiment, the soybeans were terminated by mowing on September 14, 2017, and the terminated crop was removed from the site. N fertilizer had not been applied 1.5 years prior to the start of the experiment. The site had been a combination of tillage and no‐till for several years prior to the study. The experiment was conducted at six different application timings (Table 1), with gas emissions quantified for 21 days following application, except for late fall 2017, which ended after 14 days due to soil freezing. To start each timing, eight experimental units, made up of polyvinyl chloride rings, were inserted 5 cm into the soil. All rings were placed within 2 m of each other during each timing, and the experimental units were weed free. The experiment contained two N rates (0 and 224 kg N ha^−1^), where urea, 46‐0‐0, was dissolved in 10 mL of water and sprinkled onto the soil surface within each ring. The 0 kg N ha^−1^ treatment received the same amount of water, without urea. These applications were made on the first date of each application timing. For each subsequent timing, the rings were moved to another location within the trial site.

**TABLE 1:** Average 7‐day soil temperatures, soil moisture, and water‐filled pore space (WFPS) for the surface 5 cm, the weekly amounts of rainfall and precipitation totaled over the study periods.

Timing	Date range	Soil temperature (°C)	Soil moisture (cm^3^ cm^−3^)	WFPS (cm^3^ cm^−3^)	Precipitation (cm)
Early fall 2017	Sept. 21–27	17.5	0.44 (0.12–0.52)	0.857	11
	Sept. 28–Oct. 4	15.2	0.45 (0.36–0.51)	0.877	6.5
	Oct. 5–11	11.8	0.45 (0.34–0.49)	0.877	0.2
	Average	14.8	0.45	0.870	17.7
Mid‐fall 2017	Oct. 11–18	10.9	0.32 (0.28–0.39)	0.624	0.3
	Oct. 19–25	12.2	0.32 (0.25–0.45)	0.643	0
	Oct. 26–Nov. 1	4	0.28 (0.18–0.45)	0.643	0.4
	Average	9	0.31	0.637	0.7
Late fall 2017	Nov. 1–8	3.3	0.24 (0.23–0.26)	0.468	0.7
	Nov. 9–15	2.2	0.24 (0.17–0.32)	0.468	0
	Average	2.8	0.24	0.468	0.7
Late spring 2018	May 1–8	12.6	0.37 (0.36–0.41)	0.749	1.3
	May 9–15	10.4	0.45 (0.28–0.41)	0.91	2.3
	May 16–22	14.4	0.33 (0.30–0.42)	0.668	0
	Average	12.5	0.38	0.776	3.6
Early summer 2018	May 22–29	21.9	0.27 (0.20–0.33)	0.546	0.3
	May 30–June 5	18.9	0.27 (0.19–0.32)	0.546	1.2
	June 6–12	18.5	0.26 (0.19–0.34)	0.526	0.9
	Average	19.8	0.27	0.539	2.4
Midsummer 2018	June 12–19	21.1	0.29 (0.22–0.37)	0.546	2.3
	June 20–27	18.9	0.34 (0.12–0.56)	0.465	4.2
	June 28–July 4	21	0.36 (0.26–0.68)	0.607	3.8
	Average	20.3	0.33	0.539	10.3

*Note*: The time intervals shown are 0–7, 8–14, and 15–21 days after fertilizer application. The volumetric soil moisture in parenthesis represents the range in values over each study period. This table was modified from Theis et al. (2020).

### 2.2 GHG measurements

At the start of each fertilizer application timing, the LI‐COR LI‐8100‐104 long‐term opaque chambers (8100‐104 LI‐COR) were placed over each of the eight rings. Each N treatment was replicated four times, and each chamber was programmed to pivot over the enclosure for 15 min every 4 h. This measurement approach was in accordance with others (Brugler et al., 2025; Fiedler et al., 2021; Joshi et al., 2022; Reicks et al., 2021; Thies et al., 2019, 2020). The gas within the chamber was mixed, and a vent equalized the chamber and atmospheric pressures. All eight chambers were connected to a central Picarro Cavity Ringdown Spectrometer (model G2508; Picarro Inc.) that simultaneously measured CH_4_, N_2_O, and CO_2_ concentrations every second for 15 min. Sampling periods were from 0000 to 0230 h, 0400 to 0630 h, 0800 to 1030 h, 1200 to 1430 h, 1600 to 1830 h, and 2000 to 2230 h. The sampling protocols allowed for GHG sampling at the average (0930–1030 h), minimum (0530–0630 h), and maximum air temperatures (1330–1430 h) during each day (Thies et al., 2019). The original chamber sampling order was selected at random but remained constant for each application date. Flux values were calculated based on temporal changes in the gas concentration within the chambers using version 4.01 LI‐COR SoilFluxPro software (v. 4.01; LICOR). Fluxes were corrected for the air volume within each chamber, which has a volume of 314 cm^2^. Following convention, positive values indicate that the soil was a source, whereas negative flux values showed that the soil was a sink.

**Core Ideas**

- The apparent carbon dioxide equivalent (CO_2ea_) was calculated by summing the CO_2_, N_2_O, and CH_4_ emissions.
- Urea application increased CO_2ea_ during late spring.
- During spring and summer, soils had lower CH_4_ emissions but higher CO_2_ emissions than in fall.
- The CO_2ea_ following urea application needs to be confirmed with a full‐season assessment.

The Picarro Cavity Ringdown Spectrometer factory calibration was checked with N_2_O and CO_2_ standards at the beginning and end of each experiment. The standards were purchased from Airgas Specialty Gases (Airgas USA LLC). The N_2_O standards had concentrations of 0.378 and 149 ppm, whereas the CO_2_ standards had concentrations of 99.91 and 2998 ppm. The equation between the N_2_O standard and the factory calibrations, conducted prior to and following the experiments, was *y* = 0.02 + 1.013 × (standard), *r* ^2^ = 0.99. The equation between the CO_2_ standards and the factory calibration was *y* = −0.288 + 0.994, *r* ^2^ = 1.00.

Soil moisture and temperatures for the surface 5 cm were measured in areas adjacent to the chambers using the LI‐COR LI‐8150‐205 Soil Moisture Probe (LI‐COR) and the LI‐COR LI‐8150‐203 Soil Temperature Probe (LI‐COR), respectively. During the experiments, power outages or machine failures resulted in two short gaps (September 21 to 22 and May 1 to 2). Missing information was replaced with time‐appropriate information collected from adjacent chambers having the same treatment.

### 2.3 Soil sampling

To avoid soil sampling inside the treatment rings during the study, soil samples adjacent to the chambers that were treated identically to soil within the chambers were sampled. Composite (eight cores) surface (0–15 cm) soil samples were collected on days 0, 7, 14, and 21. Samples were air‐dried, ground, sieved, and analyzed for NH_4_^+^‐N and NO_3_^−1^–N (Clay et al., 2005; Kim et al., 2008). The soil bulk density of the surface 15 cm was 1.29 and 1.34 g cm^3^ for fall samples 2017 and spring/summer samples 2018, respectively. Bulk densities and volumetric water contents were used to calculate the water‐filled pore space (WFPS), using the assumption that soil particle density was 2.65 g cm^−3^. The bulk densities were then used to convert soil gravimetric values to volumetric values.

### 2.4 Data analysis

Methane was converted to CO_2e_ by multiplying g CH_4_ m^−2^ by 27.9 and N_2_O emissions were converted to CO_2e_ by multiplying g N_2_O m^−2^ by 273 (Smith et al., 2021). The apparent carbon dioxide equivalence (CO_2ea_) was determined by summing the CO_2_, CH_4_, and N_2_O (all expressed in CO_2e_) for the 21 days following urea application. The CO_2ea_ term was used because it did not represent the full‐season emissions that could be identified as the CO_2e_.

Statistical analysis was conducted using the Agricolae package in R Studio (R Core Team, 2023). For the analysis of variance, the daily emission value from each chamber for a given application timing was conducted using a fixed effect model using the analysis of variance (aov) function in R Studio (Posit Team, 2023). Post hoc analysis was conducted using the Duncan test in the Agricolae Package of R Studio (de Mendiburu & Yaseen, 2023). When *p*‐values were <0.05, means were considered significantly different.

Correlation analysis was conducted between daily emissions and environmental values for fall and spring/summer values. For the fall correlations, information from early and mid‐fall was combined. For the spring/summer correlations, data from the late spring, early summer, and midsummer samples were combined.

## 3 RESULTS AND DISCUSSION

In 2017, soil temperatures decreased from 14.8°C in early fall to 2.8°C in late fall. Soil temperatures then increased in 2018 from 12.5°C in late spring to 19.8°C in early summer. Early summer and midsummer had similar soil temperatures (Table 1, Figures S1 and S2). These changes are expected in northern soils with a frigid climate regime. The measured soil moisture and calculated WFPS values were dependent on rainfall that varied during the study. For example, rainfall and WFPS were higher during the early fall than the mid‐ or late fall, whereas in 2018, WFPS was higher in the late spring than the early and midsummer.

### 3.1 Application date by N fertilizer rate interaction on CO_2_ and CO_2ea_

The CO_2ea_ was influenced (*p* < 0.01) by a fertilizer application date and N fertilizer rate interaction (Table 2). This interaction occurred because late spring urea application increased total CO_2ea_, but the other application dates did not. This CO_2ea_ increase during late spring was driven by a 35% increase in CO_2_ emissions (compared to unfertilized soil) in response to urea application. Conversely, urea application during midsummer reduced CO_2_ emissions by 23%. However, increased N_2_O emissions from the fertilizer during the midsummer offset the reduced CO_2_ emissions, leading to no significant difference in total CO_2_e between the fertilized and unfertilized soil.

**TABLE 2:** The effect of the fertilizer application date and N rate on CO_2ea_ derived from CO_2_, N_2_O, and CH_4_.

Application date	N rate (kg N ha^−1^)	CO_2_^a^	N_2_O	CH_4_	Total CO_2ea_
		g CO_2ea_ (ha × day)^−1^			
Early fall	0	66,578	4901	−48.5	71,431
Early fall	224	61,816	5810	−35	67,591
*p*‐value		0.67	0.5	0.4	0.77
Mid‐fall	0	42,554	1366	−45.1	43,875
Mid‐fall	224	49,482	1908	−54.3	51,336
*p*‐value		0.53	0.67	0.57	0.54
Late fall	0	11,306	120	−51.3	11,375
Late fall	224	20,639	120	−45.5	20,714
*p*‐value		0.49	0.99	0.8	0.49
Late spring	0	80,998	5403	−93.6	86,307
Late spring	224	109,463	6539	−94.9	115,907
*p*‐value		**0.01**	0.4	0.94	**0.03**
Early summer	0	111,059	987	−107	111,939
Early summer	224	89,777	2294	−64.6	92,006
*p*‐value		0.08	0.35	**0.01**	0.12
Midsummer	0	112,303	958	−90.1	113,171
Midsummer	224	86,536	3773	−64.9	90,244
*p*‐value		**0.03**	0.05	0.12	0.08
	0	76,188	2486	−72.6	78,601
	224	69,601	3407	−59.9	72,948
	*p*‐value	0.16	0.08	**0.03**	0.25

*Note*: The total CO_2ea_ is also provided.

Abbreviation: CO_2ea_, apparent carbon dioxide equivalence.

It is unclear why urea application increased CO_2_ emissions during late spring but decreased emissions in midsummer. Others have also observed higher CO_2_ emissions following soil thawing (Balewa et al., 2022; Reicks et al., 2021; Sennett et al., 2024; Su et al., 2024). One theory is that soil thawing, which normally occurs during March, can increase dissolved SOC concentrations, which can be subsequently mineralized and emitted as CO_2_ (Badewa et al., 2022; Maharjan et al., 2013). Since South Dakota soil temperatures typically remain cool through April, with minimal biological activity, the effects from thawing on CO_2_ emissions may have occurred in late spring (May 1–22). Ramirez et al. (2010) also reported an increase in CO_2_ emissions from urea applications but reported a decrease in CO_2_ emissions from non‐urea N fertilizers. These differences may be attributed to urea hydrolysis increasing the soil pH, which in turn increased dissolved SOC, followed by the mineralization and subsequent releases of the CO_2_ derived from the dissolved SOC (Clay et al., 1995; Clay et al., 1996; Curtin et al., 2016; Fender et al., 2012; Fiedler et al., 2021; Joshi et al., 2024; Liu et al., 1995; Sullivan et al., 2013).

The fertilizer application date findings suggest CO_2ea_ can be minimized by applying the N in the fall when the soil temperatures are <10°C or during the summer. Others have reported that climatic conditions can impact emissions. For example, Brugler et al. (2025) reported urea to not increase CO_2_ emissions during the dry cool year of 2021 but increase CO_2_ emissions during the wet warmer year of 2022. While there is a general perception that N losses are greater in a fall application compared to spring applied N application, these results indicate that full‐season assessments of all possible application timings are warranted.

### 3.2 Application date effect on CH_4,_ CO_2,_ and CO_2ea_ emissions

Fertilizer application timing influenced the relationships between the emitted gases. For example, in the fall and spring, a negative relationship was observed between CH_4_ and CO_2_ emissions (Table 4). This relationship indicates that increased CO_2_ (and CO_2ea_) emissions were often associated with decreased CH_4_ emissions (increasingly negative values). This was apparent during spring/summer when CO_2_ emissions were at their highest but CH_4_ emissions were at their lowest value (Table 3). During the fall, these trends reversed, and CO_2_ emissions were at their lowest and CH_4_ at their highest value. Others have reported that CO_2_ emissions increase with temperatures (Basheer et al., 2024; Buragiene et al., 2019), which we only observed during the fall. For example, CO_2_ emissions and CO_2ea_ decreased from early fall to late fall. This decrease was attributed to soil temperatures that decreased from 14.8°C to 2.8°C over this period (Tables 1 and 3). However, there were no significant differences between late spring, early summer, and midsummer, even though the soil temperature rose from 12.5°C to 20.3°C. This was also shown in Table 4 where a correlation was observed between soil temperature and CO_2_ emissions during early and mid‐fall, but not during spring and summer.

**TABLE 3:** The impact of fertilizer application timing on apparent carbon dioxide equivalence (CO_2ea_).

Application date	N rate (kg N ha^−1^)	CO_2_	N_2_O	CH_4_	Total CO_2ea_
		g CO_2ea_ (ha × day)^−1^			
Early fall		64,037b	4902a	−41.8a	69,375b
Mid‐fall		45,862c	1498b	−49.7a	47,475c
Late fall		18,482d	164b	−47.5a	18,424d
Late spring		95,061a	5465a	−94.2b	100,967a
Early summer		100,247a	1501b	−85.8b	101,831a
Midsummer		99,249a	2165b	−77.5b	101,565a
*p*‐value		**<0.01**	**<0.01**	**<0.01**	**<0.01**
	0	76,188	2486	−74.5	78,607
	224	69,601	3407	−59.9	72,954
*p*‐value		0.16	0.08	**0.03**	0.25

*Note*: Values within the same column followed by a different letter are different at *p* < 0.05. Values in bold indicate a significant main effect (*p* < 0.05) for the given variable.

**TABLE 4:** Correlation coefficients (*r*) between soil temperature (°C), average daily CH_4_ emissions (g CO_2ea_ ha^−1^), average daily CO_2_ emissions (g CO_2ea_ ha^−1^), average daily N_2_O emissions (g CO_2ea_ ha^−1^), average daily NH_3_ (g NH_3_‐N ha^−1^) emissions, soil NO_3_^−^ (kg‐N ha^−1^), and soil NH_4_^+^ (kg N ha^−1^) for fall (early fall and mid‐fall) and spring (early, mid‐, and late spring samples combined).

		WFPS	Soil temperature	CH_4_	CO_2_	N_2_O	NH_3_	Soil NO_3_^−^
**Early and mid‐fall**								
	Soil temperature	**0.49**						
	CH_4_	**0.31**	−0.10					
	CO_2_	**0.27** ^a^	**0.64** ^a^	−**0.76**				
	N_2_O	**0.75** ^a^	**0.72** ^a^	−0.01	**0.31**			
	NH_3_	−0.07^a^	**0.23** ^a^	−0.21	**0.30**	−0.09		
	Soil NO_3_^−^	**0.30**	0.16	0.19	0.00	**0.44**	0.18	
	Soil NH_4_^+^	−**0.59**	−**0.33**	−0.03	−**0.25**	−**0.51**	0.12	0.02
**Spring and summer**								
	Soil temperature	−**0.76**						
	CH_4_	**0.25**	0.05					
	CO_2_	−0.16^a^	0.13^a^	−**0.50**				
	N_2_O	**0.69** ^a^	−**0.60** ^a^	0.09	−0.14			
	NH_3_	0.10^a^	−0.13^a^	0.13	0.09	0.12		
	Soil NO_3_^−^	0.05	−0.09	**0.28**	−**0.25**	**0.44**	0.14	
	Soil NH_4_^+^	−**0.38**	**0.31**	**0.43**	−**0.26**	0.05	**0.22**	**0.63**

*Note*: Values in bold font are significant at *p* < 0.05.

Abbreviations: CO_2ea_, apparent carbon dioxide equivalence, WFPS, water‐filled pore space.

^a^ Originally reported in Thies et al. (2020).

High soil moisture levels during early fall, which were >0.85 WFPS for all 3 weeks (Table 1), may have contributed to the increased CH_4_ emissions relative to late spring (Table 3), even though these two timings had similar soil temperatures. van den Pol‐van Dasselaar et al. (1998) reported that the soil moisture range for maximum CH_4_ oxidation was between 20% and 35% (w/w), and that when soil moisture contents exceeded 50%, CH_4_ flux increased. These results were attributed to an anaerobic environment that was less favorable for methane oxidation and more favorable for methane‐producing methanogens (Bodelier & Laanbroek, 2004; Su et al., 2024). In our study, a positive correlation was shown between soil moisture and CH_4_ emissions for the fall and spring/summer application dates (Table 4).

### 3.3 N application effect on CH_4_ flux

In this study, CH_4_ emissions were 24.4% lower in the 0 than the 224 kg N ha^−1^ treatment (Table 3). These findings were attributed to the same enzyme, methane monooxygenase, catalyzing methane oxidation to methanol and ammonia oxidation to hydroxylamine (Aronson & Helliker, 2010; Arp et al., 2002). Therefore, when N is applied to the soil, methanotrophs can switch from CH_4_ to NH_4_^+^ as a substrate. When this happens, CH_4_ fluxes can increase. Evidence that this occurred was the positive correlations between CH_4_ emissions and soil NH_4_^+^ levels for the fall and spring/summer fertilizer application dates (Table 4). Aronson and Helliker (2010) suggested that this switch can occur when NH_4_^+^ fertilizer is applied at rates greater than 100 kg N ha^−1^ year^−1^.

### 3.4 Methane oxidation ability to offset N_2_O emissions

In central North America, many farms have a corn (*Z. mays*) followed by soybean (*G. max*) annual crop rotation. Our analysis suggests that during the soybean year when fertilizer is not applied, 9%–10% of the N_2_O emissions could be offset by reduced CH_4_ emissions during the early summer and midsummer (Table 3). However, it is important to note that the total CO_2ea_ offset was practically zero from methane emission reductions. In a production setting, sufficient quantities of crop residue are often produced to offset all CO_2_ emissions from the soil. For example, Clay et al. (2015) reported a net positive SOC addition over a 5‐year period under three different continuous corn management systems at this same location. These findings are important and indicate the CH_4_ oxidation and crop residue additions should be considered when calculating CO_2e_.

## 4 SUMMARY

The results from this study showed that the N fertilizer application date impacts CO_2ea_. The CO_2ea_ was higher for spring‐applied N but not for fertilizer applied at the other timings. The results from this study also showed that well‐drained agricultural soils can reduce GHG emissions by serving as a net CH_4_ sink (i.e., consuming more CH_4_ than emitting). CH_4_ oxidation was 84% greater during spring (May 1 to July 4, 2018) than during fall (September 21 to November 14, 2017). Across the N fertilizer rates and application timings, CH_4_ oxidation offset soil N_2_O emissions by 4.6% when both gases were adjusted to CO_2e_. During early and midsummer, CH_4_ oxidation offset N_2_O emissions by 9.6% when N fertilizer was not applied, and by 3.1% when N was applied. Findings from this study suggest that because fertilizer application date impacts CO_2ea_, full‐season research is warranted.

## AUTHOR CONTRIBUTIONS

**G. W. Reicks**: Data curation; formal analysis; investigation; software; writing—original draft. **D. E. Clay**: Conceptualization; data curation; formal analysis; funding acquisition; investigation; methodology; project administration; resources; supervision; validation; writing—original draft; writing—review and editing. **S. A. Clay**: Conceptualization; data curation; formal analysis; funding acquisition; investigation; methodology; project administration; resources; supervision; validation; writing—original draft; writing—review and editing. **D. R. Joshi**: Data curation; formal analysis; investigation; software. **J. Moriles‐Miller**: Data curation; investigation; methodology. **S. Westhoff**: Visualization; writing—review and editing. **S. A. Bruggeman**: Data curation; investigation; methodology.

## CONFLICT OF INTEREST STATEMENT

The authors declare no conflicts of interest.

## Supporting information



Supplementary Figure 1. A graphical depiction of the CH_4_ emission data collected during three fall timings in 2017 and three spring/summer timings in 2018. Urea was applied on the first date of application timing, which were early fall (September 21–October 11), mid‐fall (October 11–November 1), late fall (November 1–November 15), late spring (May 1–May 22), early summer (May 22–June 12), and midsummer (June 12–July 4). Each CH_4_ datapoint is an average of the four LI‐COR chambers from a given daily sampling period (i.e., from 0000 to 0230 h). There were six sampling periods per day.
Supplementary Figure 2. A graphical depiction of the CO_2_ emission data collected during three fall timings in 2017 and three spring/summer timings in 2018. Urea was applied on the first date of application timing, which were early fall (September 21–October 11), mid‐fall (October 11–November 1), late fall (November 1–November 15), late spring (May 1–May 22), early summer (May 22–June 12), and midsummer (June 12–July 4). Each CO_2_ datapoint is an average of the four LI‐COR chambers from a given daily sampling period (i.e., from 0000 to 0230 h). There were six sampling periods per day.
