# Supplementary material for: Late spring urea application increased apparent carbon dioxide equivalence emissions but fall and summer applications did not
Source: J Environ Qual. 2025 Jul 7;54(6):1737–45. doi: 10.1002/jeq2.70050 (PMC12593255; doi:10.1002/jeq2.70050)

Supplementary Figure 1. A graphical depiction of the CH_4_ emission data collected during three fall timings in 2017 and three spring/summer timings in 2018. Urea was applied on the first date of application timing, which were early fall (Sept. 21 – Oct. 11), mid-fall (Oct. 11 – Nov. 1), late fall (Nov. 1 – Nov. 15), late spring (May 1 – May 22), early summer (May 22 - Jun. 12), and mid-summer (Jun. 12 – Jul. 4). Each CH_4_ datapoint is an average of the four LI-COR chambers from a given daily sampling period (i.e. from 0000 to 0230 h). There were six sampling periods per day.


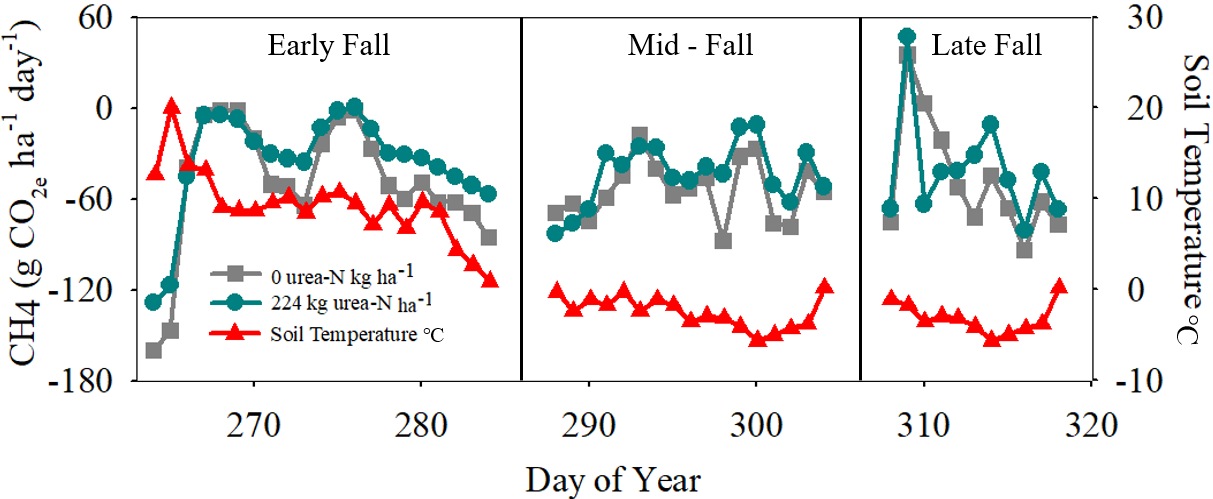


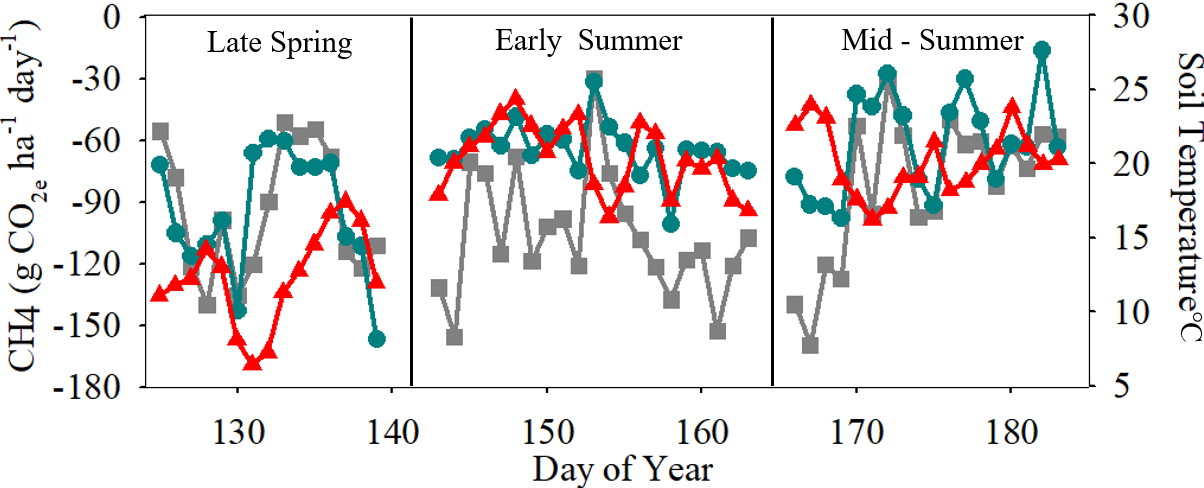


Supplementary Figure 2. A graphical depiction of the CO_2_ emission data collected during three fall timings in 2017 and three spring/summer timings in 2018. Urea was applied on the first date of application timing, which were early fall (Sept. 21 – Oct. 11), mid-fall (Oct. 11 – Nov. 1), late fall (Nov. 1 – Nov. 15), late spring (May 1 – May 22), early summer (May 22 - Jun. 12), and mid-summer (Jun. 12 – Jul. 4). Each CO_2_ datapoint is an average of the four LI-COR chambers from a given daily sampling period (i.e. from 0000 to 0230 h). There were six sampling periods per day.


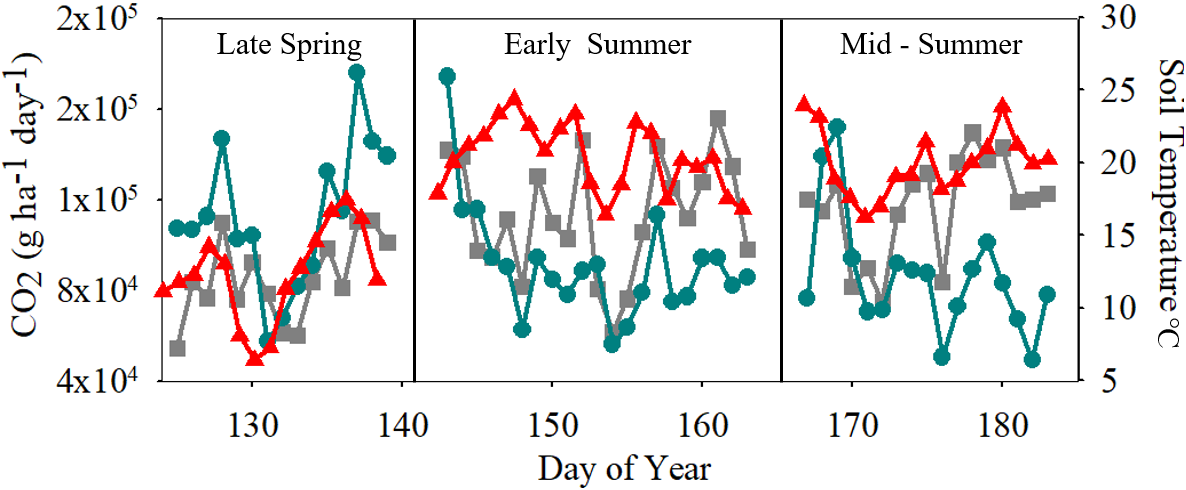


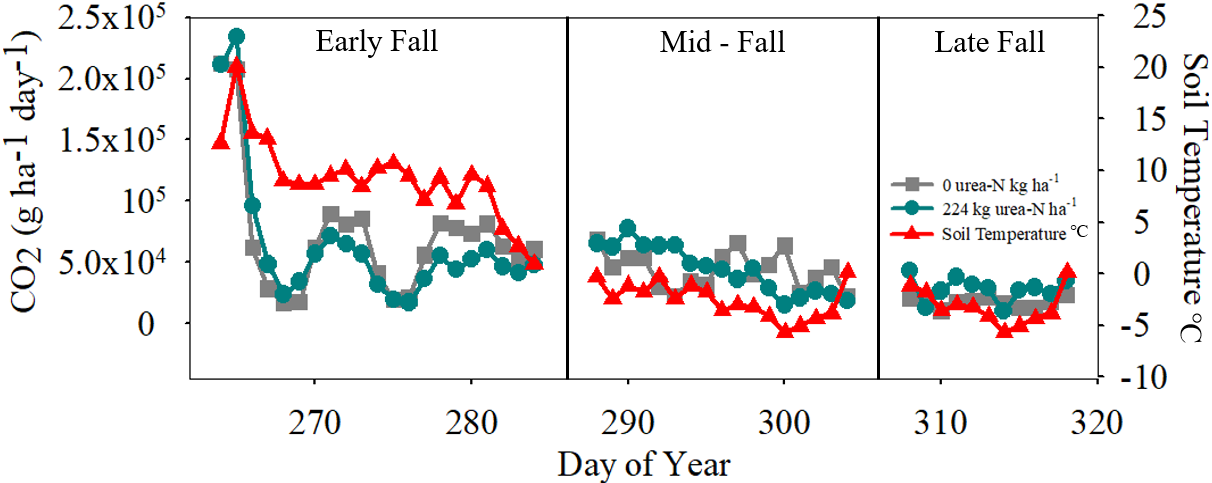

Supplement: Supplementary file 1 — Supplementary Figure 1. A graphical depiction of the CH4 emission data collected during three fall timings in 2017 and three spring/summer timings in 2018. Urea was applied on the first date of application timing, which were early fall (September 21–October 11), mid‐fall (October 11–November 1), late fall (November 1–November 15), late spring (May 1–May 22), early summer (May 22–June 12), and midsummer (June 12–July 4). Each CH4 datapoint is an average of the four LI‐COR chambers from a given daily sampling period (i.e., from 0000 to 0230 h). There were six sampling periods per day. Supplementary Figure 2. A graphical depiction of the CO2 emission data collected during three fall timings in 2017 and three spring/summer timings in 2018. Urea was applied on the first date of application timing, which were early fall (September 21–October 11), mid‐fall (October 11–November 1), late fall (November 1–November 15), late spring (May 1–May 22), early summer (May 22–June 12), and midsummer (June 12–July 4). Each CO2 datapoint is an average of the four LI‐COR chambers from a given daily sampling period (i.e., from 0000 to 0230 h). There were six sampling periods per day. [file JEQ2-54-1737-s001.docx]
